# Expanding Valve Repair in Rheumatic Heart Disease

**DOI:** 10.3389/fcvm.2021.799652

**Published:** 2022-02-04

**Authors:** Ahmed Afifi, Nairouz Shehata, Mohamed Nagi, Abdel Rahman Sultan, Magdi Yacoub

**Affiliations:** ^1^Magdi Yacoub Heart Foundation-Aswan Heart Centre, Cairo, Egypt; ^2^Department of Biomedical Engineering, Imperial College London, London, United Kingdom

**Keywords:** rheumatic mitral repair, rheumatic aortic repair, rheumatic valve imaging, rheumatic valve disease, rheumatic valve surgery

## Abstract

Rheumatic heart disease is a serious ailment with significant morbidity and mortality in endemic areas; yet, there is no agreement on indication, timing, and surgical modality for treating rheumatic valve affection. There is mounting evidence that rheumatic mitral valve repair is possible with good long-term results, less is the case with rheumatic aortic valve disease. We discuss the surgical approach for both valves emphasizing the role of multimodality imaging.

## Introduction

Affecting >1% of the population in endemic countries, rheumatic heart valve disease is responsible for the death of over 300,000 patients every year (1). Rheumatic valve disease most commonly affects the mitral valve, although around 30% of cases have aortic valve affection causing varying degrees of aortic stenosis (in 9%), regurgitation (14%), or both (6%) (2). It is a progressive disease that continues to evolve well into adulthood. There is still no agreement about the best method for treating rheumatic valve disease, with the debate of repair vs. replacement continuing.

Repair has many advantages; however, it also has its limitations and shortcomings. In this article, we review our recent developments toward perfecting the results of rheumatic valve repair with particular emphasis on the role of multimodality imaging.

## Timing and Indication

The timing of surgery in the setting of rheumatic valve disease is a topic that has not been fully addressed. Currently, the guidelines for non-rheumatic valve disease are being used to guide timing (3). Recent evidence supports rather early intervention, before the setting of irreversible myocardial damage, especially in young patients with a long, expected survival (4). It could also be added that early valve intervention, before the development of more severe rheumatic changes in the valves, could improve the likelihood of surgical repair.

## Repair vs. Replacement

Mechanical valve replacement has long been considered the classical surgical treatment for rheumatic valve disease (5). Its results are reliable in the short term with low mortality and rarely any postoperative incidence of stenosis or regurgitation. The long-term results, however, have remained far from optimal because of increased risk of thromboembolism and bleeding, both of which are further compounded by limited access to medical follow-up and anticoagulation supply (2, 6). Bioprosthesis offers a lower risk of bleeding and thromboembolism but has been shown to degenerate very quickly in young patients (5) notwithstanding the detrimental effects of prosthetic valve replacement on ventricular function, quality of life, and survival. It is, therefore, apparent that reliable valve repair would be preferred in this young population.

## The Mitral and Tricuspid Valves

Although more difficult than in degenerative mitral valve disease, rheumatic mitral valve repair is certainly possible with good outcomes, both in the short and long term, especially in young patients with isolated mitral regurgitation or stenosis (7–9). Older patients, those with mixed mitral valve pathology and calcified or diffusely thick, amalgamated valves, have generally shown less satisfying results (9). Repairing the rheumatic mitral valve, however, requires a thorough understanding of the pathophysiology of rheumatic valve disease and the dynamics of mitral valve motion during the cardiac cycle, bearing in mind that rheumatic mitral repair is unique in that it requires ensuring unobstructed flow of blood from the atrium to the ventricle, a diastolic phenomenon, not just correcting the incompetence that occurs in systole (8, 10).

**Figure 1:**
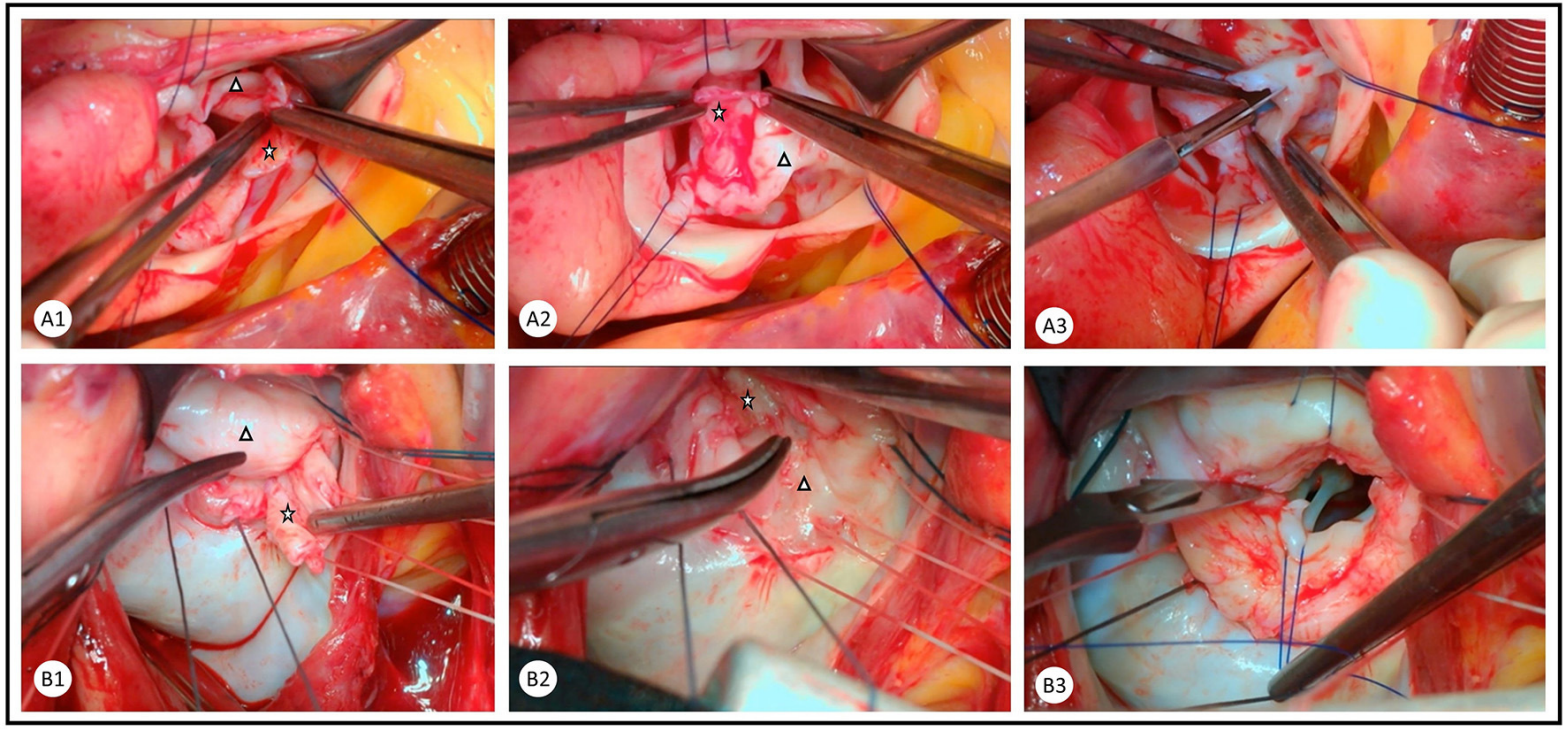
Combination of peeling and commissurotomy as an important step in the repair of rheumatic **(A)** aortic and **(B)** mitral valves. Rheumatic membrane is peeled from the ventricular surface of the aortic valve leaflets and the atrial surface of the mitral valve leaflets. The rheumatic membrane (star) is peeled from the native valve leaflet (triangle) in the **(A1)** right coronary cusp, **(A2)** non-coronary cusp, **(B1)** anterior mitral leaflet, and **(B2)** posterior mitral leaflet. Careful commissurotomy is performed with a knife and aided by forceps in the **(A3)** aortic valve and traction sutures in the **(B3)** mitral valve.

Rheumatic mitral regurgitation presents with a thin but short anterior mitral leaflet, a tethered posterior leaflet, thin elongated cords, and a dilated annulus. It is treated by following the Carpentier steps of functional segmental analysis and repair, treating the cause of incompetence, achieving a good surface of coaptation, and stabilizing the annulus (11). We have achieved this using techniques like peeling the leaflets to improve length and pliability, shortening or replacement of cords, and limiting triangular resections, or free edge application of the anterior mitral leaflet, release of secondary cords, and release of the papillary muscles from the ventricle wall and from each other. Annular stabilization and/or reduction is only achieved by a flexible posterior annuloplasty band (Figure 1).

Rheumatic mitral valve stenosis presents with varying degrees of leaflet and sub-valvular thickening, fibrosis, and calcification. The commissures are usually fused, and the annulus is rarely dilated and usually fibrotic (7). This more difficult pathology is treated by extended commissurotomy, cutting of the fused commissures in the correct plane where both sides are supported by cords, in addition, to release the commissural cords as well as the underlying papillary muscles to further enhance the opening of the valve. When the mitral leaflets are thickened, extensive peeling of the rheumatic membrane causes tangible improvement in leaflet thickness, pliability, and length. Peeling is started at the annular edge of the leaflet using a plane formed by the exit sites of the annuloplasty sutures and followed with blunt dissection till the leaflet free edge. Sharp dissection with scissors or a scalpel blade is usually necessary on the coapting surface of leaflets where the rheumatic membrane is more adherent. All thick cords are dealt by thinning, fenestration, wedge resection, or replacement. This step is particularly important in easing the diastolic opening of the valve and reducing gradient, as the diastolic inflow into the LV is an active process that requires restoration at all levels of the mitral apparatus (8). In majority of patients with isolated mitral stenosis, annuloplasty is not required.

In many patients, there is secondary tricuspid valve regurgitation, which requires treatment during operation. Not uncommonly, there is an associated primary rheumatic pathology in the tricuspid valve, causing thickening, fibrosis, and shortening of leaflets and cords. Commissural fusion could sometimes be found, causing tricuspid stenosis, which should be dealt with by commissurotomy that is usually more straightforward than that of the mitral valve. We have a low threshold for performing tricuspid valve annuloplasty, a practice that has recently been validated by a randomized trial (12).

## The Aortic Valve

Repairing the rheumatic aortic valve poses a surgical challenge. This is due to the nature of rheumatic disease, causing stiffening and shrinkage of the leaflets, which does not leave much tissue for repair (13, 14). The most readily repairable valves are those with mild to moderate stenosis or regurgitation in patients who are admitted for the repair of mitral pathology. These are corrected by sharp dissection of commissures with a scalpel and accompanied by blunt dissection to peel the rheumatic membrane from the ventricular surface of the cusps (Figure 1). This technique could be effective in both moderate aortic stenosis and regurgitation, as it leads to improved length and mobility of the leaflets (15).

In more severe cases of aortic stenosis, the previous technique could be accompanied by focal decalcification and shaving of the free edge of the leaflets. This, however, has not yielded better long-term results than aortic valve replacement with bioprosthesis, and even short-term freedom from mild to moderate aortic stenosis is not guaranteed, as residual aortic stenosis usually results from the stiff leaflets (15). Removal of the cusps and implantation of autologous pericardial leaflets with high commissural suspension (Ozaki technique) has shown very good mid-term results, but it should be considered as stent-less aortic valve replacement rather than repair (16).

Augmentation of the aortic leaflets with pericardium is also beneficial in cases with rheumatic aortic regurgitation. We prefer incising the belly of the cusps and adding a lozenge-shaped pericardial patch that would result in a larger leaflet size and a free edge of native aortic tissue. This, in our hands, has yielded better results than augmenting the cusp-free edge with a ribbon of the pericardium (15). For all the above techniques, the use of pericardium, fresh or treated, has not been optimal, neither the use of other materials, like PTFE or extracellular matrix (16–18). The use of tissue-engineered patches with optimal shape and material will expectantly be beneficial in the future for reconstruction or replacement of valve leaflets (19). For irreparable aortic valves, pulmonary autograft implantation, the Ross operation, remains the best surgical treatment modality that offers superior survival and quality of life advantage (20).

## The Essential Role of Multimodality Imaging

**Figure 2:**
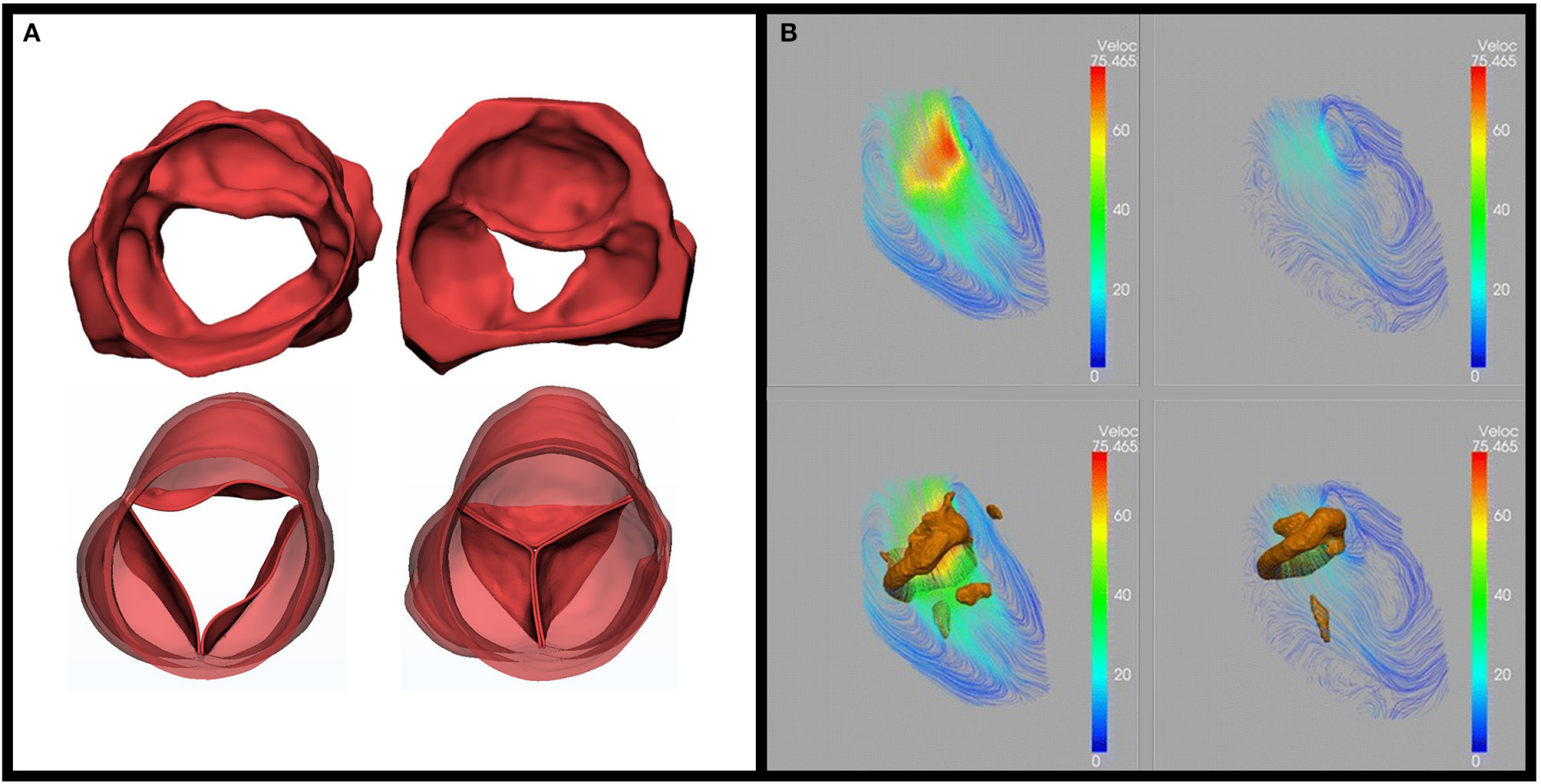
Role of multimodality imaging. **(A)** Three-dimensional 3D segmentation from computed tomography for an advanced rheumatic aortic valve unsuitable for repair (top) and after replacement (Ross operation) (bottom) in systole (left) and diastole (right). The images show shrunken and motion restricted valve leaflets preoperatively, no regurge, and excellent coaptation postoperatively adapted from Afifi et al. (15). **(B)** Use of MRI 4D flow: streamline and vortex rings. On a cross-sectional view of LV during (a) early filling and (b) late filling from Elbaz, et al. Vortex flow during early and late left ventricular filling in normal subjects: Quantitative characterization using retrospectively-gated 4D flow cardiovascular magnetic resonance and three-dimensional vortex core analysis. Journal of cardiovascular magnetic resonance (20).

Assessment of the suitability of the aortic valve for repair, guiding the type of valve conserving procedure, and importantly determining the early and long-term efficacy of the intervention depends on defining valve structure and function. This is achieved by the combination of multimodality imaging and a variety of computerized image analysis techniques. The available imaging modalities include 2- and 3-dimensional echocardiography, continuous flow Doppler, multi-slice CT, and cardiac magnetic resonance (CMR). Image analysis aims at defining the size, shape, thickness, and mobility of each component of the valve, degree of leaflet coaptation, and kinematics of the leaflets and annulus. In addition, ventriculo-arterial coupling, energy loss, and pattern of flow-through and distal to the valve are determined (Figure 2).

A novel combination of CT segmentation and four-dimensional (4D) echocardiography mesh segmentation to determine the morphodynamism of the mitral valve could provide the ideal method of assessing the functional anatomy of the valve. Studying these parameters helps in optimizing surgical techniques toward a personalized approach. Importantly, MRI-determined 4D imaging plays a major role in planning a surgical technique to maintain LV function (Figure 2) (21). This supports the concept of applying the concept of maintaining morphodynamism in cardiac surgery (22, 23).

## Conclusions and Future Direction

Techniques continue to evolve, but as it currently stands, there is no perfect repair for rheumatic valve disease. In a young and physically active population with limited access to medical care, it is important to try hard to avoid replacing these valves even if it is just a temporizing solution (24). A tailored approach to rheumatic valve disease should be followed based on patient profile and cardiac condition, with the aid of multimodality imaging (8). Equally important is to identify which valves are not suitable for reasonable valve repair, be it due to extent of valve pathology, overall cardiac condition, or patient profile.

There is a pressing need for specialized centers for the treatment of rheumatic heart valve disease where hospitals and surgeons, and volume of cases would increase the likelihood of a successful, durable, and, importantly, reproducible repair (25, 26). It is also critical to enhance facilities for postoperative follow-up of patients in terms of medical treatment, anticoagulation, ventricular function, and, importantly, quality of life.

## Data Availability Statement

The original contributions presented in the study are included in the article/supplementary material, further inquiries can be directed to the corresponding author.

## Author Contributions

AA and MY contributing to the intellectual and writing components as well as the surgical details and figures. NS, MN, and AS contributed to intellectual and writing components of the multimodality imaging section of the manuscript in addition to the relevant figures. MY guiding the intellectual direction of the manuscript, revising, and editing all sections of the work. All authors contributed to the article and approved the submitted version.

## Conflict of Interest

The authors declare that the research was conducted in the absence of any commercial or financial relationships that could be construed as a potential conflict of interest. The reviewer PP declared a shared affiliation, with one of the authors MY to the handling editor at the time of the review.

## Publisher's Note

All claims expressed in this article are solely those of the authors and do not necessarily represent those of their affiliated organizations, or those of the publisher, the editors and the reviewers. Any product that may be evaluated in this article, or claim that may be made by its manufacturer, is not guaranteed or endorsed by the publisher.
